# Measuring Environmental and Economic Performance of Air Pollution Control for Province-Level Areas in China

**DOI:** 10.3390/ijerph16081378

**Published:** 2019-04-17

**Authors:** Xiao Gong, Jianing Mi, Chunyan Wei, Ruitao Yang

**Affiliations:** 1 School of Management, Harbin Institute of Technology, Harbin 150080, China; mijianing@hit.edu.cn; 2 Department of Mathematics, Heilongjiang Institute of Technology, Harbin 150050, China; chunyan_wei@hotmail.com; 3 Center of Ultra-Precision Optoelectronic Instrument Engineering, Harbin Institute of Technology, Harbin 150080, China; ruitao.yang@hotmail.com

**Keywords:** environmental-economic efficiency (EEE), three-stage DEA model, air pollution control, province-level areas of China

## Abstract

This paper proposes an improved three-stage data envelopment analysis (DEA) model to measure the environmental-economic efficiency (EEE) of air pollution control for 30 province-level areas of China during the period of 2012 to 2016. In this model, capital, labor, and total energy consumption are the three inputs, while gross domestic product (GDP) and waste gas emissions represent the desirable and undesirable outputs, respectively. This model allows the weights of economic growth and environmental protection to be adjusted as needed by policymakers; the model is adopted to evaluate the effects of government measures on environmental protection and economic growth. Ultimately, the effects from environmental factors and statistical noise are excluded from the EEEs of local governments and the managerial efficiencies are calculated. The results simultaneously reflect the local performance of air pollution control and economic development, which can be used to clarify the ranking of provinces nationwide.

## 1. Introduction

Clean air is fundamental for human and ecosystem health as well as ensuring future development in the world. Given the rapid economic development and industrialization, governments face an increasing number of issues caused by air pollution [1]. Air pollution refers to harmful or excessive quantities of substances, including gases, particulates, and biological molecules, in the atmosphere, whose properties and duration may damage human health and other living organisms. Governments, especially in developing countries, have been forced to make trade-offs between their energy supply stability, environmental issues, and economic development [2]; this is also the situation in China [3].

**Figure 1:**
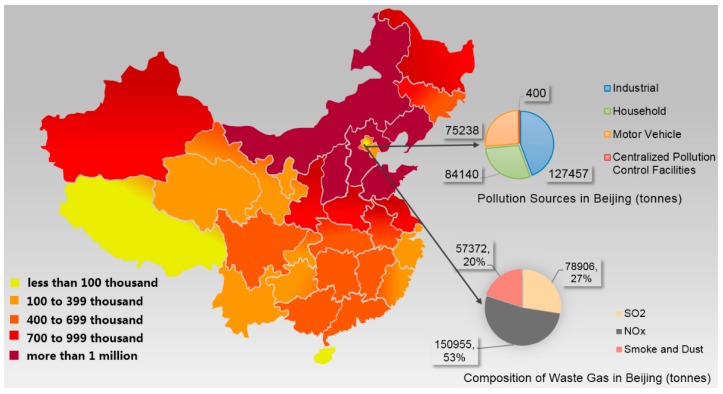
Waste gas emissions in Chinese districts in 2014 (tons) [8].

**Figure 2:**
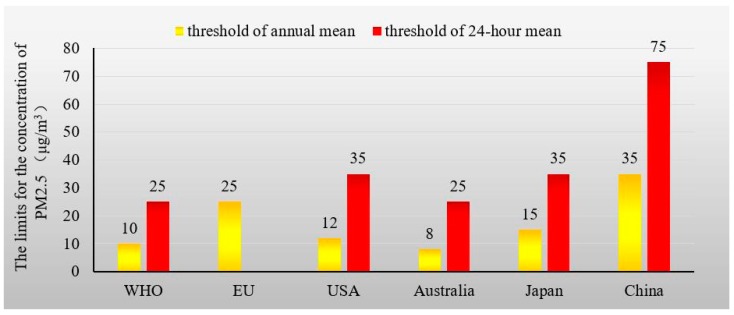
International limits for PM_2.5_ (μg/m^3^) concentrations [8]. WHO represents the World Health Organization. EU represents the European Union.

In recent years, China has encountered severe air pollution, especially in winter. Widespread and prolonged smog has severely impacted public health and national economic progress [4,5]. The poor air quality and resultant poor visibility have resulted in traffic problems, and children have been warned to stay indoors to decrease their exposure to pollutants [6,7]. The northern and eastern regions have experienced particularly severe air pollution as a result of waste gas emissions [8]. Figure 1 shows the air pollution levels in 2014. In certain northeastern Chinese cities, such as Shenyang and Harbin, the peak concentrations of PM_2.5_ (i.e., fine particulate matter with a diameter of under 2.5 micrometers) exceeded 1000 μg/m^3^, far above the upper limits for all international standards, as shown in Figure 2 [9]. However, many municipalities hope to create a positive reputation and an attractive city label (e.g., “eco-city” and “natural oxygen bar”) to attract investors and secure support from higher government tiers [10,11]. These goals will never be achieved if concerns about the balance between air pollution and economic development are not resolved.

Scholars have conducted many studies on China’s gas emissions. However, most of these studies focus on the emission efficiency of greenhouse gases [12,13,14,15,16,17]. The issue of waste gas resulting in air pollution has attracted relatively little attention, and the focus has generally turned to China’s industrial sectors. Within these studies, air pollutants have been separated, with studies investigating the impact of economic activity and municipal households on PM_2.5_ pollution in certain regions, including the Beijing-Tianjin-Hebei Region, the Yangtze River Delta, and other prosperous areas [18,19,20]. In addition to smoke and dust, which are types of fine particulate matter, the other pollutants monitored in China’s current official air quality grading system are sulfur dioxide (SO_2_) and nitrogen oxides (NO_X_). Wu et al. [21] and Ge et al. [22] used NO_2_ as an environmental index to evaluate the environmental efficiency of China’s industry in different provinces. SO_2_ and industrial dust have been studied to assess environmental performance in relation to China’s growth in total factor productivity and in the auto manufacturing sector [23]. By contrast, Yang and Li [24] used total industrial waste gas as an assessment index to evaluate 39 industrial sectors because scholars continue to treat the industry as the dominant economic component.

However, there is no specific substance that harms human health or threatens social development, and industrial emissions are not the only source of waste gas [25,26]. Table 1 shows the main sources and respective percentages of these waste gases for 2015. Although industrial emissions represent a large portion of total waste gas, pollution from other sources cannot be ignored when evaluating environmental performance. In particular, because this study aims to consider both urban quality of life and social sustainable development to provide an integrated evaluation of economic and environmental performance, any pollutant from any source should be taken into account. Moreover, the role of local government is indispensable, although it has been ignored in previous studies in this field. Accordingly, this study aims to provide research results that can increase the effectiveness of government measures. Furthermore, this study simultaneously considers economic development and environmental protection and takes into account the role of local governments because local governments do not expect to reduce waste gas emission at the expense of economic growth. Therefore, the purpose of this study is threefold. First, this study aims to contribute to the literature by proposing an improved index of environmental-economic efficiency (EEE) to simultaneously measure economic and environmental performance. Second, a three-stage data envelopment analysis (DEA) model is adopted to explore the impact of local governments’ measures on air pollution control. Third, from an empirical research perspective, the results of this paper present an accurate assessment of managerial efficiency of air pollution control in China’s province-level areas by using the total waste gas emission as an environmental index. 

This paper is organized as follows. Section 2 reviews the literature on waste gas and DEA models. Section 3 introduces a three-stage DEA model with undesirable outputs that evaluates the environmental and economic performance of different provinces in China from a public governance perspective. Section 4 calculates the EEE and analyzes the results by combining the model with MATLAB programming. Finally, the conclusion and policy recommendations are provided in the last section.

## 2. Literature Review

### 2.1. Research on Air Pollution Control

Due to increasing concern about air pollution within both public opinion and academic circles, numerous studies have been conducted in the field of waste gas, some of which are introduced in the above section. This section presents previous work from other perspectives.

One stream of research focuses on estimating the economic losses caused by air pollution. For example, the detailed economic cost of chemical pollution was calculated by analyzing the damage caused to buildings in France [28]. Similarly, losses in Nigeria caused by air pollution were evaluated by analyzing the impact on local people’s health and the resulting medical costs [29]. Other scholars have collected data on Chinese patients suffering from acute respiratory and other relevant diseases and demonstrated that air pollution increased total medical costs and reduced social welfare [30]. Welsch [31] estimated the monetary value of air pollution by comparing its impact on people’s happiness along with its impact on income. In the field of public policy, increases in total social welfare have been studied to compensate for the loss caused by climate change and severe weather. In this study, loss refers to the damage to people’s health, buildings, and farm crops resulting from air pollution [32].

Studies have also focused on the control and treatment measures of air pollution. The earliest study dates to the early 20th century and the concept of a Pigouvian tax [33]. Pigou [33] noted that the adverse side effects of market activity, such as environmental pollution, should be covered by a product’s market price rather than being borne by the innocent people who live near a pollution source. Therefore, it was suggested the government impose a tax to fill the gap between the private cost of the activity and the social cost of the negative externalities. In recent years, many scholars have conducted further research in this field. An increasing number of mathematical models have been used to estimate the cost that should be covered by taxation and other market mechanisms. Spadaro and Ari [34] built a function to calculate the cost per kilogram of air pollution and then designed a corresponding tax plan. Chinese scholars developed a dynamic analysis model and considered the features of industrial development, the profitability of local enterprises, energy structure, and the features of air pollution. The effects of different tax policies on sulfur emission reduction in different districts were determined [35]. Subsequently, a PSM-DID model was used to evaluate the effects of different emission rights trading policies on sulfur emission reduction [36].

Recently, an increasing amount of research has focused on the efficiency of emissions reduction and the control of waste gases. Fujii et al. [37] analyzed 10 Chinese industrial sectors over 12 years and noted that end-of-pipe treatment could reduce the amount of fine particulate matter discharged to 35% of its current level. Lai et al. [38] applied the directional distance function to determine China’s provincial shadow price for different energy sources, such as coal, petroleum, natural gas, and electricity, in the 15 years since 1988. The results showed that both the national total efficiency of energy consumption and provincial technical efficiency declined in this period. However, most studies in the field prefer the DEA model. This method will be introduced in the next section.

### 2.2. Measuring Environmental Performance with the Data Envelopment Analysis (DEA) Model

In studies on efficiency in the control and reduction of air pollution, various indicators have been proposed to measure environmental performance. A general approach is to collect data on various sub-indicators that present the characteristics of interest, measure particular features of the research object, and then establish a complete environmental performance assessment system [39]. However, it is difficult to obtain scientific guidelines for improving performance through a comparison with other objects with high efficiency. The DEA method is capable of addressing this drawback because it uses specified inputs and outputs to obtain the calculation results, which means it is necessary to aggregate the environmental sub-indicators. In addition, the DEA model does not propose hypotheses or parameters before building a function, which minimizes interference from subjective conceptions. Therefore, the DEA model has become increasingly popular and widely accepted in the field of efficiency research.

The DEA model was initially proposed by Charnes et al. [40] and Charnes and Cooper [41], and used to evaluate the efficiency of a set of comparable objects, which are defined as decision-making units (DMUs). DEA is used to identify those DMUs that produce the highest number of desirable outputs while requiring the fewest inputs. Mathematical programming is used to calculate a set of efficiency scores that are equal to or less than 1. The efficiency of a DMU is the ratio of its weighted outputs to its weighted inputs; thus, if efficiency equals 1, the corresponding DMU is considered efficient. DEA makes it possible to optimize the objective DMU because the weights of the inputs and outputs can be determined in the calculation process. Therefore, the DEA method has been widely accepted in studies evaluating performance. In the field of environmental performance, Zhou et al. [42] performed a complete and detailed literature review on the application of DEA that included more than 100 studies and discussed the selection of DEA models.

Clearly, pollutant emissions provide an important index for evaluating environmental performance. Accordingly, undesirable outputs, such as CO_2_ and waste gas emissions, should be considered in the DEA method. There are two main approaches to accomplishing this analysis. The first, represented by Seiford and Zhu [43] and Yeh et al. [44], translates these pollutant emissions into inputs based on data translation and then uses the conventional DEA method to determine the DMUs’ efficiencies. Other studies treat the undesirable outputs as having weak disposability [45,46]. These undesirable outputs are treated as byproducts of the desirable outputs and cannot be reduced without cost. A nonradial DEA model and its foundation, the Malmquist index, were developed by Zhou et al. [47], who evaluated the change in environmental performance for the OECD (Organization for Economic Cooperation and Development) member countries over time.

A majority of previous studies focused on measuring the efficiency of pollutant emissions or energy consumption while ignoring contemporaneous economic development. According to Wu et al. [39], Zhou et al. [48] was the first study to take into account both environmental performance and economic development. Based on this work, Wu et al. [39] fixed the model and used it to evaluate environmental and economic performance for the APEC (Asia-Pacific Economic Cooperation) countries. This paper hence adopts the model in Wu et al. [39], with minor modifications, and other two stage processing to isolate the effect from environmental factors and statistic noise.

All of the studies noted above have attributed the slack variable, which is the gap between the inputs/outputs of efficient and inefficient DMUs, to the inefficient management of DMUs. However, Fried et al. [49,50] argued that the drivers of the slack variable should be divided into environmental effects, statistical noise, and managerial inefficiency. This categorization implies that the influence of the external operating environment and statistical noise should be eliminated if more accurate managerial effectiveness of DMUs is needed [51]. This process is very important in the case of China, especially when the research target is the public administration’s influence on environmental and economic performance in the provinces, because there are major differences among the provinces in aspects, such as population density, economic structure, local culture, and ethnic issues. Taking the Tibet Autonomous Region as an extreme example, it has a province-level administration, but much of its economic and social data is not captured even in the China Statistical Yearbook published by the NBS (National Bureau of Statistics of China). Therefore, this paper removes environmental effects and statistical noise by adopting the method proposed in Fried et al. [49] with minor modifications.

In conclusion, numerous studies have focused on waste gases from the perspective of their control and the economic losses that they cause. However, few studies have simultaneously focused on both economic and environmental performance or examined these factors at the provincial level in China. Additionally, most studies evaluating waste gas emission performance attribute slack to managerial inefficiency and ignore the impacts of external environmental effects and noise. The removal of any effects of environmental factors and statistical noise is necessary to determine the performance of local governance on the economy and air environment in China’s provinces. Therefore, this study first adopts the latest environmental DEA model advanced by Wu et al. [39] to determine the EEE of provinces and then removes the impacts of external environmental effects and noise through stochastic frontier analysis (SFA) and a second application of the DEA. These methods are introduced in detail in the next section.

## 3. Materials and Methods

As mentioned in Section 2.2, the DMUs’ efficiency scores can be calculated with the one-stage DEA model. However, the result of this efficiency score is not only determined by the managerial efficiency, but also the environmental factors and statistical noise [49,50]. To eliminate the influence from environmental factors and statistical noise and estimate the managerial efficiencies of each DMU more precisely, the three-stage DEA model was proposed in [49,50]. The principle of the three-stage DEA method is presented in this section.

### 3.1. DEA for Environmental-Economical Efficiency Calculation

#### 3.1.1. Conventional DEA Model with Undesirable Output

Consider a DMU that uses *n* inputs, $X=(x_{1},\cdots ,x_{N})$, to produce outputs, including *m* desirable outputs, $Y=(y_{1},\cdots ,y_{M})$, and *j* undesirable outputs, $U=(u_{1},\cdots ,u_{J})$. This production process can be depicted by a production technology, *T*:

(1) T={(x,y,u):x can produce (y,u)}.

Given both the undesirable and the desirable outputs included in the above production process, two assumptions for *T* were proposed by Faere and Grosskopf [45]:

1. The outputs are weakly disposable, i.e., if (x,y,u)∈T, and 0≤θ≤1, then (x,θy,θu)∈T;

2. The desirable outputs and the undesirable are null-joint, i.e., if (x,y,u)∈T, and *u* = 0, then *y* = 0.

Assumption 1 means that the costs of undesirable outputs cannot be independently reduced and may cause a proportional reduction of the desirable outputs. Assumption 2 means that all outputs are produced jointly, and undesirable outputs cannot be eliminated unless the whole production process is stopped. Thus far, the production technology, *T*, has been defined clearly, but it still cannot be directly employed to evaluate performance in case studies.

Faere and Grosskopf [45] further proposed an environmental DEA technology that is a piecewise linear programming technology under constant returns to scale. For a $DMU_{k}\left(k=1,2,\cdots ,K\right)$, its inputs and outputs can be represented as $x_{k}=(x_{1k},\cdots ,x_{Nk})$, $y_{k}=(y_{1k},\cdots ,y_{Mk})$ and $u_{k}=\left(u_{1k},\cdots ,u_{Jk}\right)$, respectively. Then, *T* can be defined as follows:

(2) $$\begin{array}{cc} T=\{(x,y,u): & \sum_{k=1}^{k}z_{k}x_{nk}\leq x_{n},\;n=1,\ldots ,N\\ & \sum_{k=1}^{k}z_{k}y_{mk}\geq y_{m},\;m=1,\ldots ,M\\ & \sum_{k=1}^{k}z_{k}u_{jk}=u_{j},\;j=1,\ldots ,J\\ & z_{k}\geq 0,\;k=1,\ldots ,k\}. \end{array}$$

#### 3.1.2. Calculation of Economic-Environmental Efficiency

Conventional environmental DEA technology focuses on exploring either the minimum level of pollutant emission or the maximum level of economic growth. However, it is impossible for governments to consider only environmental protection or economic performance in the policymaking process. Scholars thus hope to find a method to help balance the two. The following two models are combined to evaluate the two types of performance simultaneously. Model 1 is the undesirable outputs-oriented DEA model proposed in Tyteca [52]:

(Model 1)

(3) $$\lambda_{}^{*}=\min\lambda ,$$

(4) $$s.t.\;\sum_{k=1}^{k}z_{k}x_{nk}\leq x_{n0},\;n=1,\cdots ,N\left(input\right),$$

(5) $$\sum_{k=1}^{k}z_{k}y_{mk}\geq y_{m0},\;m=1,\cdots ,M\left(desirable\;outputs\right),$$

(6) $$\sum_{k=1}^{k}z_{k}u_{jk}=\lambda u_{j0},\;j=1,\cdots ,J\left(undesirable\;outputs\right),$$

$$z_{k}\geq 0,k=1,\cdots ,K.$$

In the model, “*s.t.*” represents “subject to” and Equations (4)–(6) are the constraint conditions. In comparison with Equation (2), in which the undesirable outputs are not allowed to be adjusted, Model 1 introduces an adjustment factor of λ to the undesirable outputs. In this case, the desirable output and undesirable output become the given data from observation. Additionally, the adjustment factor of λ should be calculated with Model 1. In other words, the model aims to determine the minimum level of undesirable outputs for each DMU (Equations (3) and (6)), while holding the new inputs at or below the current level (Equations (4)) and the new desirable outputs at or above the current level (Equation (5)). However, in the application of these conventional DEA models, two different DMUs often have the same efficiency score of 1, although one of the two has fewer inputs and more outputs than the other. To avoid this case and determine the efficient DMU, Cooper et al. [53] focused on the slacks and proposed the slack-based measure (SBM) model. Based on the DEA-SBM model, Wu et al. [39] improved the previous study and evaluated the economic-environmental index with Model 2:

(Model 2)

(7) $$\min\rho_{}^{*}=\frac{1-\frac{1}{N}\sum_{n=1}^{N}\frac{s_{n}^{-}}{x_{n0}}}{1+\frac{1}{M}\left(\sum_{m=1}^{M}\frac{s_{m}^{-}}{y_{m0}}+\frac{s_{uk}^{-}}{\lambda_{}^{*}u_{0}}\right)},$$

(8) $$s.t.\;\sum_{k=1}^{k}z_{k}x_{nk}+s_{nk}^{-}=x_{n0},\;n=1,\cdots ,N,$$

(9) $$\sum_{k=1}^{k}z_{k}y_{mk}-s_{mk}^{+}=y_{m0},\;m=1,\cdots ,M,$$

(10) $$\sum_{k=1}^{k}z_{k}u_{jk}+s_{uk}^{-}=\lambda_{}^{*}u_{j0},\;j=1,\cdots ,J,$$

$$z_{k}\geq 0,k=1,\cdots ,K.$$

In this model, ordinary inputs and outputs are adopted. $s_{nk}^{-}$ and $s_{uk}^{-}$ represent the surpluses of the inputs and undesirable outputs, respectively, compared with the efficient DMU. $s_{mk}^{+}$ represents the shortage of desirable outputs. The λ^*^ in Equation (10) is the optimal objective of Model 1 that allows the undesirable outputs to be adjusted into an optimized value in Model 2. That is, these DMUs are efficient in terms of the pollutant emission performance. Within these constraints, Model 2 is primarily used to evaluate economic performance based on a DEA-SBM model. ρ^*^ is the objective value of this linear programming problem and represents economic efficiency under the assumption that the corresponding DMU is environmentally efficient. If ρ^*^ = 1 and $s_{n}^{-}=s_{u}^{-}=s_{m}^{+}=0$, the DMU is both economically and environmentally efficient.

In conclusion, λ^*^ as calculated by Model 1 is the environmental efficiency value, and ρ^*^ from Model 2 is the economic efficiency value under the assumption of λ^*^ = 1. On this basis, the index of environmental-economic efficiency (EEE) is proposed to measure both economic and environmental performance simultaneously. In this paper, EEE is calculated by the weighted sum in Equation (11). In actual public governance, policymakers can set the weights depending on the focus of their administration:

(11) $$EEE=\omega_{1}\lambda_{}^{*}+\omega_{2}\rho_{}^{*}.$$

### 3.2. Stochastic Frontier Analysis (SFA)

#### 3.2.1. Theory of SFA

The second stage of this research uses SFA. In this stage, the research focus is the slack variables, which are the difference between the actual and the optimized inputs/outputs of the efficient DMU. The slacks were initially treated as reflecting managerial inefficiency according to the theory of the DEA model. However, Fried et al. [49] noted that this initial inefficiency should be divided into managerial inefficiency, environmental effects, and the statistical noise caused by measurement errors. Therefore, the research target in the second stage is to decompose the input slack variables calculated in the first stage into the above three effects. To achieve this goal, SFA is used to regress the input slacks against environmental variables and statistical error. In SFA regression models, the dependent variables are all of the input slacks, which are calculated by Equation (12), and the regressions take the form of Equation (13):

(12) $$S_{ni}=x_{ni}-X_{n}z\geq 0,$$

n=1,2,⋯,N, i=1,2,⋯,I,

(13) $$S_{ni}=f\left(h_{i};\beta_{n}\right)+v_{ni}+\mu_{ni},$$

where *S_ni_* represents the *i*-th input slacks for the *n*-th DMU, *X_n_z* is the optimal outcome of *x_n_* in the efficient DMU, $f\left(h_{i};\beta_{n}\right)$ is the deterministic feasible slack frontier with the observable environmental variable, *h_i_*, and its parameter, *β_n_*, and *v_ni_* and *μ_ni_* represent the statistical noise and managerial inefficiency, respectively. To estimate the regression, Equation (13), these variables must meet the following two assumptions:

1. *v_ni_* and *μ_ni_* are normally distributed. $v_{ni}∼N\left(0,\sigma_{vn}^{2}\right)$, and $u_{ni}\geq 0,u_{ni}∼N^{+}\left(\mu_{n},\sigma_{un}^{2}\right)$;
2. *v_ni_*, *μ_ni_*, and *h_i_* are distributed independently of each other.

Under these assumptions, the parameters that need to be estimated in the regression function are *β_n_*, *μ_n_*, *σ_vn_*^2^, and *σ_un_*^2^.

In Equation (13), the environmental effect is directly expressed as the deterministic feasible slack frontier, $f\left(h_{i};\beta_{n}\right)$. Since stochastic noise exists in the environmental effect, the SFSF (stochastic feasible slack frontier) is expressed as $S_{ni}=f\left(h_{i};\beta_{n}\right)+v_{ni}$, which corresponds to the minimum slack in a noisy environment because of the nonnegative *μ_ni_*. Therefore, the rest of Equation (13), *μ_ni_*, represents the slack in excess of the SFSF. Thus far, managerial inefficiency, *μ_ni_*, has been separated from other effects.

#### 3.2.2. Adjustment of Inputs Based on SFA’s Result

The original input is adjusted by the result of the SFA to obtain the real managerial efficiency. The objective of this process is to ensure that all producers are at the same level and to avoid the unfairness caused by environmental effects and statistical noise before reusing the DEA model. In other words, if production proceeds in an unfavorable environment, the relevant DMU will be disadvantaged in the first evaluation because the assessment method in the first stage does not take the environment with noise into account. According to Fried et al. [49], to level the playing field, the relevant advantaged environmental factor and good luck in statistical noise should be transformed into part of the inputs and then added to the original inputs. The transformation can be expressed as follows:

(14) $$\begin{array}{l} x_{ni}^{A}=x_{ni}+\left[\max\left(f\left(h_{i};{\hat{\beta }}_{n}\right)\right)-f(h_{i};{\overset{\wedge }{\beta }}_{n})\right]+\left[\max\left(v_{ni}\right)-v_{ni}\right],\\ i=1,2,\cdots ,I;n=1,2,\cdots ,N, \end{array}$$

where $x_{ni}^{A}$ and $x_{ni}^{}$ represent the adjusted input and the original, respectively. The second item of Equation (14) is the adjustment for environmental effects, which locates all production processes in a common working environment. The last item is the adjustment for stochastic noise, which locates all production processes in a common context and eliminates concerns about the factor of luck. With these adjustments, if a production process is in a beneficial environment and/or is subject to good luck, a relatively small value will be added to the original inputs for the corresponding DMU. Similarly, if a production process is in a disadvantageous environment and/or is subject to bad luck, a relatively large value will be added to the original inputs.

To implement Equation (14), *v_ni_* should be estimated by stripping stochastic noise from the residuals of the SFA regression models in Equation (13). According to Fried et al. [50], the method proposed by Jondrow et al. [54] can be adopted to accomplish this objective. However, in Jondrow’s work, the formula for stripping stochastic noise was based on a production function and the composed error items are represented by ε=ν−μ. Since the SFA regression model in this paper adopts a cost function in which the composed error items are ε=ν+μ, the conditional estimator formula is fixed as follows:

(15) $$E\left(\mu |\varepsilon \right)=\sigma^{*}[\frac{\phi \left(\lambda \frac{\varepsilon }{\sigma }\right)}{\Phi \left(\frac{\lambda \varepsilon }{\sigma }\right)}+\frac{\lambda \varepsilon }{\sigma }],$$

where $\sigma^{*}=\frac{\sigma_{u}\sigma_{v}}{\sigma },\sigma =\sqrt{\sigma_{u}^{2}+\sigma_{v}^{2}}$ and $\lambda =\sigma_{u}/\sigma_{v}$. Stochastic noise can be estimated according to Equation (16):

(16) $$E\left[v_{ni}|v_{ni}+u_{ni}\right]=s_{ni}-f\left(h_{i};\beta_{n}\right)-E\left[u_{ni}|v_{ni}+u_{ni}\right],$$

where $E\left[u_{ni}|v_{ni}+u_{ni}\right]$ represents the conditional estimator for managerial inefficiency. Equation (16) accordingly provides a conditional estimator for stochastic noise. According to Assumption 1 presented in Section 3.2.1, $E\left[v_{ni}|v_{ni}+u_{ni}\right]$ depends on $\left(\beta_{n},\sigma_{vn}^{2},\sigma_{un}^{2},\mu_{n}\right)$ as well as $E\left[u_{ni}|v_{ni}+u_{ni}\right]$. Therefore, $\beta_{n}$ is estimated to reflect the extent to which each environmental variable affects the *n*th input slack, and the remaining $\sigma_{vn}^{2},\sigma_{un}^{2},\mu_{n}$ are estimated to characterize the separate impacts of stochastic noise and managerial inefficiency on the slack.

### 3.3. Managerial Efficiency Evaluation by DEA with the Adjusted Inputs

The third stage is a repetition of the DEA, but with the input from the first stage replaced by $x_{ni}^{A}$, which is obtained in the second stage. The adjusted input counteracts the effect of the environmental factors and statistical noise. The output is thus the evaluation of pure managerial efficiency.

## 4. Results

This section aims to calculate the EEE of air pollution control for 30 province-level areas of China, which reflects the performance of air pollution control and economic development simultaneously. However, there are major differences among the provinces in aspects, such as population density, energy intensity, local government’s influence, economic structure, and even ethnic issues. The initial EEE calculated by Model 2 in the first stage thus includes the effect of environmental factors and statistic noise. Therefore, it is necessary to use SFA to decompose the inefficiency calculated in the first stage into managerial inefficiency, environmental effects, and the statistical noise in the second stage. The original inputs are then adjusted by the results of the SFA to ensure that all provinces experience the environmental effects at the same level and to avoid the unfairness caused by statistical noise. The third stage is a repetition of Model 2 with the adjusted inputs and the local governments’ managerial efficiencies worked out finally.

In this section, input variables, output variables, and environmental variables are defined. All raw data are selected from the China Statistical Yearbook and the China Energy Statistical Yearbook, which are published by the National Bureau of Statistics of China (NBS). To evaluate local governments’ administrative performance in controlling and preventing air pollution, the three-stage calculation is completed as follows.

### 4.1. Stage 1: The Initial DEA Performance Evaluation with Original Inputs

#### 4.1.1. Variables and Data Selection

In [55], 20 studies were reviewed on the evaluation of environmental performance with the DEA model, and production was defined such that each DMU uses capital, labor, and energy to produce desirable outputs and inevitable pollutions. In this paper, each Chinese province is treated as a producer, and its public administrative performance is evaluated. The production is accordingly defined such that each province puts in capital, labor, and energy to gain the desirable output of GDP (gross domestic product) and undesirable output of air pollution. The input variables thus include the following:

1. Capital. By convention in related research, the capital stock from annual fixed asset investment in different provinces is chosen as the capital input factor in our study. Based on the popular perpetual inventory accounting method, capital stock, *K_n,t_*, from the annual fixed asset investment of the province, *n*, in the year, *t*, can be estimated as follows:
  (17) $$K_{n,t}=K_{n,t-1}+I_{n,t}-D_{n,t}=\left(1-d_{n,t}\right)K_{n,t-1}+I_{n,t},$$
  where *K_n,t_*_ − 1_ represents the capital stock from the fixed asset investment of a specific province, *n*, in the year, *t* − 1. *I_n,t_* is the newly added fixed asset investment of the province, *n*, in the year, *t*, which adopts the gross fixed capital formation (GFCF) value published by the NBS. *D_n,t_* is the fixed asset depreciation of province *n* in year *t*, while *d_n,t_* represents the depreciation rate of the fixed assets of province *n* in year *t*. According to Zhang’s studies on the estimation of China’ s provincial capital stock, the depreciation rate was calculated to be 9.6% [56,57]. This value has been widely adopted in studies involving China’s provincial capital stock, such as in [13,55,58,59]. In this study, the provincial capital from 2000 to 2016 can thus be estimated, and the data over the last 5 years are adopted in this paper.
2. Labor. In this paper, the input labor consists of employed persons in urban units, persons engaged in private enterprise, and self-employed individuals from both urban and rural areas. The data source is the China Statistical Yearbook [60].
3. Total energy consumption. As one of the most important input factors, the total energy consumption of each province is utilized in this paper to evaluate the provincial EEE. All data come from the China Energy Statistical Yearbook [61].

The output variables include the following:

1. GDP. This study uses each provincial GDP as the desirable output [60].
2. Waste gases. In this paper, annual total waste gases are studied as an undesirable output. These gases comprise nitrogen oxides, sulfur dioxides, and smoke and dust in the air. All the data come from the China Statistical Yearbook 2013 to 2017 [60].

#### 4.1.2. Environmental-Economic Efficiency (EEE)

The model presented in Section 3.1 is used to calculate the original EEE, and the calculation is completed by MATLAB programming. In addition to MATLAB programming, many software products can be used to calculate the DEA model, such as DEAP, DEA-Solver, and MaxDEA. Although these programs calculate quite similar results, there are differences [24]. To obtain explicit results for this new model, this study develops a particular MATLAB algorithm that realizes EEE-oriented calculations. In the calculation, the economy and the environment are given the same weight: In Equation (4), $\omega_{1}=\omega_{2}=0.5$. Table 2 presents the overall EEE scores for 22 provinces, 4 municipalities directly under the central government and 4 autonomous regions. Tibet is absent from the evaluation due to a lack of data. Table 2 presents the results for the EEE scores to the second decimal point, while the rank shows the minor differences among these fairly similar figures.

**Figure 3:**
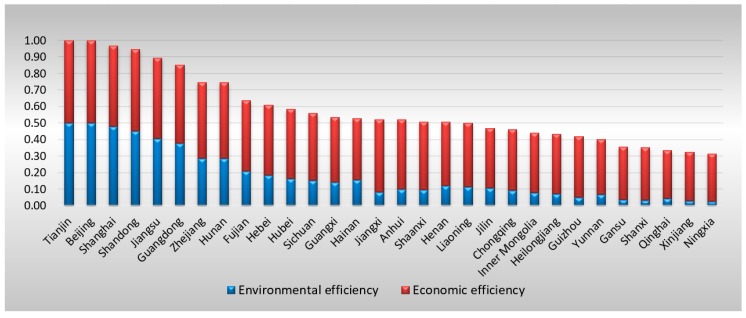
Average original EEE for provinces (2012–2016) in Stage 1. Source: Drafted by authors.

As shown in Table 2, Beijing and Tianjin were identified as efficient provinces for five consecutive years when performance in terms of GDP growth and air pollution reduction were given the same weight. To provide a general understanding of these provinces’ performance for both local economic development and control over waste gas emissions, Figure 3 shows their average EEEs from 2012 to 2016 and their rankings. It can be clearly seen that low environmental efficiency caused provinces to perform worse in the rankings than low economic efficiency.

### 4.2. Stage 2: Applying SFA to Decompose Stage 1 Slack

#### 4.2.1. Variables and Data Selection

In the second stage, the SFA method is used to separate environmental effects and statistical noise from the initial inefficiency. The input slacks calculated in the first stage are regressed over the observed environment factor, and the initial input of the DEA model is then adjusted for the result of the regression. The adjusted inputs thus level the playing field. That is, the first stage of the performance evaluation does not take into account certain advantageous factors for provinces, such as a relatively favorable environment or relatively good luck. The inputs of these provinces are adjusted upward, and the amounts of the adjustment depend on the extent to which they benefit from these factors. This process is completed using Frontier Version 4.1 software.

Panel data covering the years from 2012 to 2016 are selected. In previous studies on the emissions efficiency of waste gases and CO_2_, GDP per capita and the value-added percentages of GDP by industry sector and for the service sector were explored as explanatory environmental variables [39,62]. In addition to these variables, energy intensity was studied in [39,63]. As provincial policymakers and regulators, the influence of local governments is critical to policy enforcement and the performance of waste gas emissions [64]. In this research, government influence is denoted as the proportion of local governments’ expenditure to GDP. Therefore, there are five environmental variables: GDP per capita (GPC), the value-added percentage of GDP by the industry sector (VPGIS), the value-added percentage of GDP by the service sector (VPGSS), energy intensity (EI), and government influence (GI).

#### 4.2.2. Application of Stochastic Frontier Analysis (SFA)

Before applying SFA to regress the slacks calculated by Equation (5) in the first stage against the observable environmental factors, it is necessary to resolve the possible problem of multi-collinearity among the above explanatory variables. Accordingly, Pearson correlation coefficients for the variables of interest are studied and presented in Table 3.

From this table, it can be seen that GPC is highly correlated with VPGIS (0.65), EI is highly correlated with GI (0.69), VPGIS is highly correlated with VPGSS (0.83), VPGSS is highly correlated with GPC (0.65) and VPGIS (0.83), and GI is highly correlated with EI (0.69). To resolve this problem, the highly correlated explanatory variables are replaced by orthogonal residuals, which is a method used by Wu et al. [39] and Hong et al. [65]. The implementation procedure in this study is as follows.

Step 1. Regress the VPGSS over the GPC and the VPGIS to obtain the residual of VPGSS (Res_VPGSS).

Step 2. Regress the GI over the EI to obtain the residual of GI (Res_GI).

Step 3. Run SFA with GPC, EI, VPGIS, Res_VPGSS, and Res_GI rather than GPC, EI, VPGIS, VPGSS, and GI. The result from the SFA is given in Table 4.

In the SFA results shown in Table 4, the LR test of one-sided errors is determined to be significant at a 1% confidence level by look-up tables [66], which indicates that there is an obvious composite structure in the error term of Equation (5). It is therefore necessary to apply the SFA method to the input slacks and the improved environmental variables. In addition, the results summarized in Table 4 show that the operating environment influences the input slacks to different degrees. For example, GPC is shown to be negatively related to the slack of capital, as are VPGIS and VPGSS, while EI and GI are positively correlated with it. Therefore, the impacts of the operating environment and stochastic noise must be decomposed so that all producers (in this study, the provinces) can be revaluated in the same environment and with similar luck.

### 4.3. Stage 3: DEA Performance Evaluation with Adjusted Inputs

The input variables are adjusted by the results of the SFA in the last stage, and the EEE is recalculated by Model 2 in this stage. EEE represents only the provincial governments’ managerial performance in terms of both economic development and control over waste gas emissions given the same stochastic noise and after eliminating effects from environmental factors, such as the strength of local governments and respective economic characteristics. The final results and ranks are shown in Table 5.

**Figure 4:**
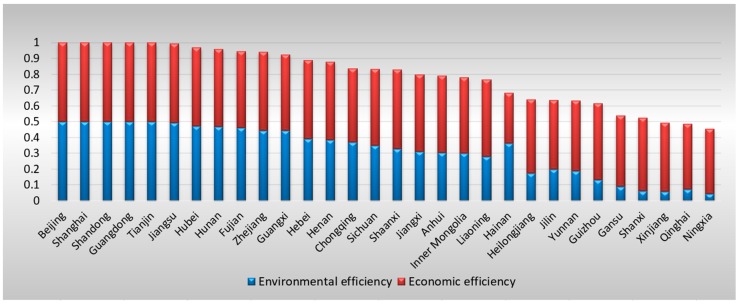
Average final EEE for provinces (2012–2016) in Stage 3.

Table 5 presents the overall final EEE scores for these province-level areas. After adjusting the inputs of the DMUs that are in a disadvantaged environment or that have been unlucky, three more areas are identified as efficient DMUs for the five consecutive years—Shanghai, Shandong, and Guangdong—while only Beijing and Tianjin were identified in the first stage. To obtain a general understanding of these provinces’ final performance in terms of both local economic development and control over waste gas emissions, Figure 4 shows their average EEEs from 2012 to 2016 and their rankings. It can be clearly seen that the rankings are completely different from the original rankings in Stage 1; for example, Jiangsu Province becomes the sixth most efficient area after averaging across the years. In addition, the contribution of environmental efficiency to EEE is underestimated in the first stage by comparing Figure 3 and Figure 4.

Different analysis results are obtained when exploring a specific province’s economic and environmental performance during the five years. For example, Hebei Province’s original EEE as calculated by Stage 1 dropped from number 6 to number 18, while the final EEE calculated by Stage 3 has an inverted U-shaped line. Hainan Province’s original ranking remained in the top half for all five years, while its final EEE ranked in the second half. Hainan is the only area whose final economic efficiency decreased after adjusting the original input.

## 5. Discussion 

This study expands a classical model with the aim of measuring the economic and environmental performance of province-level areas in China for local governments’ public administration. Because it offers the advantage of estimating the managerial efficiency of local governments, this study can be used for the following purposes:

First, it provides more accurate environmental performance. Performance in terms of control over air pollution as calculated by the conventional DEA model does not reflect only the public managerial efficiency of the local government because the amount of waste gas emissions is also affected by environmental factors, such as the local economic structure, population, and government strength. Thus, SFA is necessary to separate the effects of the operating environment from statistical noise. In the empirical study, by comparing Figure 3 and Figure 4, it can be seen that the environmental performance of many areas is seriously underestimated if it is not adjusted with the SFA. For example, after adjustment, the environmental efficiencies of Anhui, Jiangxi, Henan, Hubei, Guangxi, Chongqing, and Shaanxi show a more significant increase than the economic efficiencies.

Second, this approach clarifies the position of a specific area nationwide. In empirical research, Hainan Province is a good example because of its distinctive economic structure. Hainan is an important province that is famous for tourism, with an annual VPGSS that exceeded 50% from 2013 to 2016. Hainan ranked in the first half for the entire study period in the first calculation by the DEA model under the assumption that economic growth and environmental protection are weighted the same. However, after the input variables were adjusted to compensate for those areas in a disadvantaged environment, Hainan’s ranking dropped to the bottom half, which means that it did not perform as well as it did in the estimation in the conventional study. Compared with the majority of provinces, Hainan’s economic and environmental performance could be more effective.

Finally, this approach enables the statistical analysis of the change in a certain area’s environmental and economic performance. Certain provinces’ EEE changes substantially after eliminating the effects of environmental factors, stochastic noise, and the variation tendencies of a certain area over time. Taking Hebei Province as an example, its EEE score calculated by the original DEA model declined annually, dropping from number 6 in 2012 to number 18 in 2016. However, its ranking showed a symmetric inverted-U shape during this period, which means that in the middle years, the government’s work was more efficient than in 2012 and 2016. Therefore, this study provides an approach to estimate the change in a certain area and helps local governments become more efficient in balancing economic growth and environmental protection.

## 6. Conclusions

This study proposed an improved three-stage DEA model to measure the EEEs of province-level areas in China for the period of 2012 to 2016. The EEE index takes into account simultaneously the economic development and air pollution control. Since it seems impractical that formulating policies based only on environmental aspects while ignoring concerns about economic growth, Model 2 was used to calculate the EEE, which attempts to capture a balance between economic growth and air protection. This model allows the weights of economic growth and environmental protection to be adjusted as needed so that policymakers can make a tradeoff between them. Because the provinces evaluated possess significant heterogeneity in their economic characteristics, SFA was then used in the second stage to isolate managerial inefficiency from both environmental effects and statistical noise. In the third stage, the inputs were adjusted, and Model 2 was used again to re-evaluate the performance of the provinces, and the managerial efficiency of local government was calculated ultimately. The empirical results from 30 of China’s province-level areas showed that the three-stage DEA model is beneficial to measure local governments’ accurate managerial performance on air pollution control and to analyze the change in a certain area’s environmental and economic performance.

## Figures and Tables

**Table 1:** Main sources of waste gas in China’s regular air quality grading system.

Waste Gas (Percentage)	Main Source of Waste Gas (Percentage)
Sulfur dioxides (SO_2_, 35.4%)	Industrial production processes (83.7%)
	Municipal household (16.0%)
	Centralized pollution control facilities (0.1%)
Nitrogen oxides (NO_X_, 35.3%)	Industrial production processes (63.8%)
	Municipal household (3.5%)
	Motor vehicles (31.6%)
	Centralized pollution control facilities (0.1%)
Smoke and dust (29.3%)	Industrial production processes (80.1%)
	Municipal household (16.2%) Motor vehicles (3.6%) Centralized pollution control facilities (0.1%)

^1^ Source: Ministry of Ecology and Environment of the People’s Republic of China [27].

**Table 2:** Original EEE of 30 Chinese province-level areas from 2012 to 2016.

Area	2012		2013		2014		2015		2016	
	EEE	Rank	EEE	Rank	EEE	Rank	EEE	Rank	EEE	Rank
Beijing	1.00	1	1.00	4	1.00	2	1.00	3	1.00	4
Tianjin	1.00	2	1.00	5	1.00	3	1.00	2	1.00	2
Hebei	1.00	6	0.55	12	0.54	13	0.50	14	0.46	18
Shanxi	0.40	24	0.37	27	0.35	27	0.34	26	0.31	27
Inner Mongolia	0.50	21	0.46	22	0.43	23	0.42	25	0.39	24
Liaoning	0.54	19	0.53	16	0.52	17	0.53	12	0.38	25
Jilin	0.52	20	0.47	20	0.47	20	0.44	24	0.43	21
Heilongjiang	0.43	23	0.41	23	0.45	22	0.47	21	0.40	22
Shanghai	0.95	7	0.88	6	1.00	1	1.00	1	1.00	1
Jiangsu	1.00	5	0.74	8	1.00	6	1.00	4	0.73	5
Zhejiang	0.73	8	1.00	2	0.76	7	0.62	8	0.63	7
Anhui	0.58	13	0.54	13	0.52	16	0.50	15	0.46	16
Fujian	0.62	11	0.67	9	0.67	8	0.65	6	0.57	10
Jiangxi	0.57	15	0.53	17	0.52	15	0.49	16	0.49	12
Shandong	1.00	3	1.00	1	1.00	5	1.00	5	0.72	6
Henan	0.55	18	0.51	18	0.50	19	0.48	17	0.47	15
Hubei	0.58	12	0.59	11	0.57	11	0.58	10	0.59	9
Hunan	0.69	9	0.77	7	0.65	9	0.61	9	1.00	3
Guangdong	1.00	3	1.00	3	1.00	4	0.65	7	0.60	8
Guangxi	0.56	16	0.54	14	0.54	12	0.55	11	0.48	13
Hainan	0.57	14	0.54	15	0.53	14	0.51	13	0.49	11
Chongqing	0.47	22	0.46	21	0.46	21	0.46	22	0.46	19
Sichuan	0.67	10	0.59	10	0.58	10	0.48	20	0.46	17
Guizhou	0.38	26	0.39	24	0.42	24	0.46	23	0.44	20
Yunnan	0.38	27	0.38	25	0.38	25	0.48	18	0.39	23
Shaanxi	0.56	17	0.51	19	0.51	18	0.48	19	0.47	14
Gansu	0.39	25	0.37	26	0.36	26	0.33	27	0.32	26
Qinghai	0.35	28	0.34	29	0.34	28	0.33	28	0.30	28
Ningxia	0.33	30	0.34	30	0.32	30	0.30	30	0.28	29
Xinjiang	0.35	29	0.34	28	0.34	29	0.31	29	0.28	30

**Table 3:** Pearson correlation for variables before SFA.

Pearson Correlation, *n* = 150					
Pro > |r|: Rho = 0					
	GPC	EI	VPGIS	VPGSS	GI
GPC	1	–0.4478	–0.2519	0.6522	–0.4230
		<0.0001	0.0009	<0.0001	<0.0001
EI	–0.4478	1	0.2082	–0.3003	0.6906
	<0.0001		0.0053	0.0001	<0.0001
VPGIS	–0.2519	0.2082	1	–0.8330	–0.1707
	0.0009	0.0053		<0.0001	0.0184
VPGSS	0.6522	–0.3003	–0.8330	1	–0.0461
	<0.0001	<0.0001	<0.0001		0.0148
GI	–0.4230	0.6906	–0.1707	–0.0461	1
	<0.0001	<0.0001	0.0184	0.0148	

**Table 4:** Stochastic frontier estimation results (standard errors in parentheses).

Independent Variables	Input Slack		
	Capital	Labor	Energy Consumption
Constant	11.05 ** (5.13)	2.60 (4.30)	50.11 *** (8.48)
GPC	−0.91 ** (0.44)	−4.28 *** (0.18)	−4.51 *** (0.73)
EI	0.27 (0.37)	3.782 ** (1.55)	1.32 * (0.75)
VPGIS	−4.17 ** (1.91)	0.21 (0.59)	5.47 * (2.74)
Res_VPGSS	−6.50 * (4.46)	−3.65 ** (1.67)	14.82 * (7.62)
Res_GI	0.75 (0.82)	16.66 *** (6.03)	2.87 (4.65)
σ^2^	3.60 *** (0.98)	50.32 *** (15.16)	16.09 *** (4.86)
λ	0.78 *** (0.07)	0.94 *** (0.02)	0.72 *** (0.10)
Log-likelihood function	−222.80 ***	−342.94 ***	−342.27 ***
LR test of one-sided error	76.43 ***	94.52 ***	22.34 ***

* Significant at the 10% level; ** significant at the 5% level; *** significant at the 1% level or better.

**Table 5:** Final EEE of 30 Chinese province-level areas from 2012 to 2016.

Area	2012		2013		2014		2015		2016	
	EEE	Rank	EEE	Rank	EEE	Rank	EEE	Rank	EEE	Rank
Beijing	1.00	1	1.00	3	1.00	6	1.00	1	1.00	5
Tianjin	1.00	8	1.00	10	1.00	4	1.00	8	1.00	6
Hebei	0.73	16	1.00	9	1.00	9	1.00	9	0.71	16
Shanxi	0.53	25	0.51	27	0.56	27	0.53	26	0.49	27
Inner Mongolia	1.00	6	0.80	17	0.73	21	0.73	19	0.64	24
Liaoning	0.64	20	0.66	19	0.89	18	1.00	4	0.65	23
Jilin	0.61	22	0.62	22	0.67	22	0.62	25	0.66	20
Heilongjiang	0.57	23	0.54	23	0.65	23	0.80	16	0.65	22
Shanghai	1.00	5	1.00	2	1.00	3	1.00	3	1.00	1
Jiangsu	1.00	7	1.00	4	0.97	14	0.99	12	1.00	2
Zhejiang	1.00	3	0.77	18	1.00	7	0.93	13	1.00	3
Anhui	0.66	18	1.00	8	0.92	16	0.71	21	0.68	19
Fujian	0.90	15	0.94	13	1.00	1	1.00	11	0.89	10
Jiangxi	0.65	19	1.00	5	1.00	2	0.65	24	0.68	18
Shandong	1.00	4	1.00	1	1.00	5	1.00	6	1.00	7
Henan	1.00	10	1.00	6	0.81	19	0.80	15	0.79	11
Hubei	1.00	12	0.95	12	0.92	17	1.00	5	0.97	9
Hunan	0.90	14	0.89	15	1.00	13	1.00	10	1.00	8
Guangdong	1.00	2	1.00	11	1.00	8	1.00	7	1.00	4
Guangxi	0.96	13	0.90	14	1.00	11	1.00	2	0.77	12
Hainan	0.62	21	0.63	21	0.79	20	0.69	22	0.68	17
Chongqing	1.00	9	0.88	16	0.92	15	0.67	23	0.72	15
Sichuan	0.72	17	1.00	7	1.00	9	0.72	20	0.72	14
Guizhou	0.53	26	0.53	24	0.64	24	0.73	18	0.65	21
Yunnan	0.53	27	0.53	25	0.62	25	0.86	14	0.63	25
Shaanxi	1.00	11	0.65	20	1.00	12	0.74	17	0.74	13
Gansu	0.54	24	0.53	26	0.59	26	0.51	27	0.52	26
Qinghai	0.48	29	0.47	29	0.52	29	0.49	28	0.46	29
Ningxia	0.44	30	0.46	30	0.49	30	0.44	30	0.44	30
Xinjiang	0.49	28	0.49	28	0.54	28	0.48	29	0.46	28
